# Ghrelin rapidly elevates protein synthesis in vitro by employing the rpS6K-eEF2K-eEF2 signalling axis

**DOI:** 10.1007/s00018-022-04446-4

**Published:** 2022-07-16

**Authors:** Alexander V. Zhdanov, Anna V. Golubeva, Martina M. Yordanova, Dmitry E. Andreev, Ana Paula Ventura-Silva, Harriet Schellekens, Pavel V. Baranov, John F. Cryan, Dmitri B. Papkovsky

**Affiliations:** 1 School of Biochemistry & Cell Biology, University College Cork, Cavanagh Pharmacy Building, College Road, Cork, Ireland; 2 Department of Anatomy & Neuroscience, University College Cork, Cork, Ireland; 3 Belozersky Research Institute of Physico-Chemical Biology, Lomonosov Moscow State University, Moscow, Russia; 4 Shemyakin-Ovchinnikov Institute of Bioorganic Chemistry RAS, Moscow, Russia; 5 APC Microbiome Institute, University College Cork, Cork, Ireland; 6 Present Address: School of Biomolecular and Biomedical Science, University College Dublin, Dublin 4, Ireland

**Keywords:** eEF2 phosphorylation, Translation elongation, Ghrelin receptor GHS-R1α, Cell signalling, De novo protein production

## Abstract

Activated ghrelin receptor GHS-R1α triggers cell signalling pathways that modulate energy homeostasis and biosynthetic processes. However, the effects of ghrelin on mRNA translation are unknown. Using various reporter assays, here we demonstrate a rapid elevation of protein synthesis in cells within 15–30 min upon stimulation of GHS-R1α by ghrelin. We further show that ghrelin-induced activation of translation is mediated, at least in part, through the de-phosphorylation (de-suppression) of elongation factor 2 (eEF2). The levels of eEF2 phosphorylation at Thr56 decrease due to the reduced activity of eEF2 kinase, which is inhibited via Ser366 phosphorylation by rpS6 kinases. Being stress-susceptible, the ghrelin-mediated decrease in eEF2 phosphorylation can be abolished by glucose deprivation and mitochondrial uncoupling. We believe that the observed burst of translation benefits rapid restocking of neuropeptides, which are released upon GHS-R1α activation, and represents the most time- and energy-efficient way of prompt recharging the orexigenic neuronal circuitry.

**Supplementary Information:**

The online version contains supplementary material available at 10.1007/s00018-022-04446-4.

## Introduction

Ghrelin, a 28-amino acid peptide (aka ‘hunger hormone’), is produced mostly by specialised cells of the gastrointestinal tract [1] and secreted into the bloodstream in a rhythmical food intake-dependent and circadian manner [2, 3]. Ghrelin interacts with GHS-R1α receptor on the surface of a large variety of cells and activates numerous signalling circuits that control feeding behaviour, nutrient processing and metabolism [4–7]. GHS-R1α-positive neurons of the arcuate nucleus in the hypothalamus release NPY, AgRP and GABA, which cooperatively modulate the orexigenic effect of ghrelin [8]. Once neurotransmitters are secreted, their intracellular pools should be rapidly restored, and accelerated local translation of pre-existing mRNA molecules emerges as an efficient way to fulfil this task [9].

Upon ghrelin binding, GHS-R1α-dependent cascades of phosphorylation trigger key signalling pathways, which increase the rate of metabolic and bio-synthetic processes, e.g. PI3K/AKT/GSK-3β, PI3K/AKT/mTOR and Raf-1/MEK/ERK1/2 cascades [10–14]. At the same time, ghrelin induces elevation of cellular Ca^2+^ levels and sequential stimulation of Ca^2+^/CaM-dependent kinases that, among other factors, activate AMPK via Thr172 phosphorylation [15]. AMPK, in turn, down-regulates energy-demanding processes and counterbalances pro-synthetic pathways [16]. Thus, acting via raptor and TSC1/2, AMPK inhibits mTOR-dependent translation on the initiation and elongation levels [15]. The involvement of antagonistic mTOR and AMPK pathways in mRNA translation [17] and the complexity of initiation machinery [18] suggest that effects on translation initiation induced by ghrelin might be highly sensitive to physiological and cell signalling contexts.

Likewise, eEF2 kinase (eEF2K), a potent modulator of translation elongation and an effective regulator of protein synthesis [19], represents a ‘battle field’ for competitive signalling cascades activated by ghrelin [20]. On one hand, Ca^2+^- and CaM-dependent auto-phosphorylation of eEF2K at Thr348 and Ser500 increases the affinity of kinase to CaM and enhances its activity [21]. Similar effect has AMPK-driven phosphorylation of Ser398 and Ser491/Ser492 (in humans) [22]. On the other hand, p38δ MAPK-driven phosphorylation of Ser359, as well as mTORC1-specific phosphorylation of Ser78 and Ser396, reduces CaM binding and decreases kinase activity of eEF2K; phosphorylation of eEF2K at Ser366 by mTOR- and ERK1/2-dependent kinases of the ribosomal protein S6 (p70 S6K and p90 RSK, respectively) inactivates eEF2K [20]. Further adding on to the complexity of regulatory network, eEF2K levels may also change rapidly in response to a range of stress conditions via decreased eEF2K mRNA translation and active protein degradation [23, 24].

Although several novel targets of eEF2K have been recently identified in vitro [25], its unique specificity towards eEF2 phosphorylation at Thr56 is broadly accepted [26]. When phosphorylated at Thr56, eEF2 loses the capacity to translocate the tRNAs from A to P site of the ribosome, which is a key step in translation elongation [27]. Along with initiation, elongation dictates the efficiency of protein production. Hence, in light of the involvement of ghrelin signalling and eEF2 in obesity, diabetes and carcinogenesis [28–32], the control of eEF2 phosphorylation upon activation of GHS-R1α emerges as an attractive therapeutic strategy. Recently, we have demonstrated that ghrelin treatment decreases p-eEF2 levels in HEK293 cells overexpressing GHS-R1α [33]. However, to the best of our knowledge, mechanisms of ghrelin-dependent eEF2 de-suppression and effects of GHS-R1α activation on the rate of protein synthesis have not been studied.

Here we hypothesise that ghrelin can modulate mRNA translation rate in GHS-R1-positive cells and that reduced eEF2 phosphorylation can facilitate protein production de novo. To test the hypothesis and examine the underlying mechanisms, we conducted systematic analysis of de novo protein production rate and eEF2 phosphorylation state, as well as factors regulating eEF2K activity in resting and metabolically stressed cells upon activation of GHS-R1α by ghrelin.

## Methods

### Materials

Amersham™ ECL™ Prime Western blotting reagent was from GE Healthcare Life Sciences (Waukesha, WI); pre-made acrylamide gels, running and transfer buffers were from GeneScript (Piscataway, NJ). Genopure plasmid midi kit, complete protease inhibitor cocktail tablets and phosSTOP phosphatase inhibitor tablets were from Roche (Mannheim, Germany). Fura Red AM, Oregon Green™ 488 BAPTA-1 (OGB-1) AM, Immobilon™-P PVDF Transfer Membranes, Lipofectamine® 2000, OptiMEM I, BCA™ Protein Assay kit, Click-iT^®^ HPG Alexa Fluor 594 Protein Synthesis Assay Kit (cat. C10429), DAPI (4´,6-diamidino-2-phenylindole) and RIPA buffer were from Thermo Fisher Scientific (Rockford, Ill). Ghrelin, okadaic acid (OKA, an inhibitor of PP2A; IC50 = 0.2–1 nM) and BRD7389 (an inhibitor of rpS6 RSK; IC50 = 1.2, 1.5 and 2.4 μM for RSK3, RSK1 and RSK2, respectively) were from Tocris (UK). SNAP-Cell^®^ 647-SiR, pSNAPf Vector, SNAP-Cell® Block, *Gaussia* luciferase and BioLux® *Gaussia* Luciferase Assay Kit were from NEB (Ipswich, MA). m^7^GTP-agarose beads (γ-aminohexyl-m^7^GTP-agarose) were from Jena Biosciences (Jena, Germany). RNeasy® Plus Universal Mini Kit was from Qiagen (Venlo, Netherlands); High-Capacity cDNA Reverse Transcription Kit was from Applied Biosystems (Waltham, MA) and SensiFAST SYBR Lo-ROX kit—from Bioline (London, UK). Antibodies were from: Alomone Labs, Israel (ghrelin receptor, № AGR-031); Becton Dickinson, NJ (eIF4G2 aka NAT1, № 610,742); Cell Signalling Technology, MA (CREB, № 1385; phospho-CREB (Ser133), № 9198; mTOR, № 2972; phospho-mTOR (Ser2448), № 2971; phospho-ERK1/2 (Thr202/Tyr204), № 9101; eEF2, № 2332; phospho-eEF2 (Thr56), № 2331; eEF2K, № 3692; phospho-eEF2K (Ser366), № 3691; eIF4G1, № 2498; 4E-BP1, № 9452; eIF2α, № 5324; phospho-eIF2α (Set51), № 3398; p-AKT (Ser473), № 4060; tuberin/TSC2, № 4304; phospho-TSC2 (Thr1462), № 3617; rpS6, №2217; phospho-rpS6 (Ser235/236), № 2271, PDCD4, № 9535); ECM Biosciences, KY (eEF2K Phospho-Regulation Antibody Sampler Kit, № EK6910, which includes antibodies against Ser78, Ser359, Thr348, Ser398, Ser500 in eEF2K and eEF2K C-terminus); Millipore, CA (ERK1/2, № 06–182); Proteintech, IL (AKT, № 10,176–2-AP); Sigma, MO (α-tubulin №T5168, HRP-conjugated anti-rabbit and anti-mouse antibodies, № A1949 and A0168); Thermo Fisher Scientific, MA (anti-rabbit Alexa Fluor 555-conjugated, № A-21428). Dulbecco's modified Eagle medium (DMEM) high glucose, DMEM without glucose, L-glutamine and pyruvate, DMEM without L-glutamine, sodium pyruvate, L-methionine and L-cystine, geneticin (G418), ampicillin, cycloheximide (CHX), PF4708671 (an inhibitor of p70 rpS6 kinase S6K1 isoform; IC50 = 160 nM), m^7^GTP, Bradford reagent, l-cysteine, l-glutamine and all the other reagents were from Sigma-Aldrich-Millipore. Plasticware was from Sarstedt (Ireland), MatTek (Ashland, MA) and Greiner Bio One (Frickenhausen, Germany).

### Tissue culture and cell treatment

HEK293 stably expressing GHS-R1α-EGFP (HEK293^GHS-R1α-EGFP+^) [34] and embryonic mouse hypothalamus N38 and N41 cells (Hypo E-N38 and Hypo E-N41, Cedarlane Laboratories, Burlington, Canada) were cultured in DMEM supplemented with 10% FBS, 100 U/ml penicillin/100 µg/ml streptomycin (P/S), non-essential amino acids (NEAA) and 10 mM HEPES, pH 7.2 (complete DMEM).

For protein isolation, cells were seeded at 4.5 × 10^5^–1 × 10^6^ per well of 6-well plate, grown for 24–48 h in the same medium and then for 12–14 h either in low serum DMEM (1% FBS, no NEAA, no antibiotics) or serum-free DMEM (NEAA, no FBS, no antibiotics) prior to treatments. In these two similar pre-incubation conditions, cell responses to ghrelin treatment were practically identical, and they were applied in all experiment, unless stated otherwise. For a ‘metabolic stress’ control, cells were pre-incubated in the media supplemented with galactose instead of glucose; glutamine and pyruvate were provided in both cases to support oxidative phosphorylation (OxPhos); media were supplemented with 1% glucose-free FBS dialysed against 0.9% NaCl.

For confocal imaging, cells were seeded at 2 × 10^4^ cells per cm^2^ on glass-bottom mini Petri dishes (MatTek, Ashland, MA). Cell staining with Ca^2+^ indicators Fura Red (4 µM, 70 min) and OGB-1 (4 µM, 90 min) was followed by two washes and 30 min de-esterification.

See Results section for further details on cell treatment with ghrelin and other compounds.

### De novo protein production analysis

Translation rate was examined by measuring de novo production of 3 reporter proteins (a–c) and total protein production (d) in HEK293^GHS-R1α-EGFP+^ cells.

Plasmids encoding reporters were purified using standard protocols and Genopure Plasmid Midi Kit (Sigma). Plasmid DNA was delivered to the cells using a one-day Lipofectamine^®^ 2000-based protocol. For this, 2 × 10^6^ cells were seeded on 10 cm Petri dishes (Sarstedt), grown for 16 h and transfected in 5 ml of OPTIMEM for 4 h using 100 ng DNA and 0.4 ml Lipofectamine per 1 cm^2^. OPTIMEM was replaced with complete DMEM, and after 4 h incubation trypsinised (several Petri dishes were combined if needed), counted and re-seeded at 4 × 10^4^ cells per well on 96-well plates (Fast-FT, *G*Luc), at 7.5 × 10^5^ cells per well on 6-well plates (SNAP), or at 1.5 × 10^4^ cells/cm^2^ on MatTek glass bottom dishes (Fast-FT, SNAP).

#### Fluorescent timer

Cells transfected with a plasmid encoding fluorescent timer Fast-FT [35] were grown for up to 24 h in complete DMEM and for 12 h in low serum DMEM. Dual blue/red fluorescence was measured on: 1) an Olympus FV1000 confocal microscope at 420–520/560–660 nm (excitation at 405/543 nm, respectively) using glass-bottom mini Petri dishes and 2) a multimode plate reader FlexStation 3 (Molecular Devices, Sunnyvale, CA) with similar excitation/emission settings using 96-well plates. For a plate reader experiment, cells transfected with Fast-FT for 4 h on a 9 cm Petri dish were incubated for 3 h in complete DMEM, trypsinised, resuspended in DMEM (1% FBS) and seeded at 7.5 × 10^4^ cells per well on a 96-well plate. CHX treatment was used to inhibit translation in control dishes/wells. The ratio of blue fluorescence signals in ghrelin- and mock-treated cells at different time-points was normalised to the corresponding ratio of red fluorescence signals. Plate reader showed less data variability and was considered more suitable for quantitative analysis.

#### Gaussia luciferase activity

Cells transfected with the plasmid encoding *Gaussia* luciferase [36] were grown for up to 24 h in complete DMEM and for 12 h in low serum DMEM (or serum-free DMEM supplemented with NEAA). To analyse secreted luciferase activity, medium was replaced with 100 µl of fresh low serum DMEM (or serum-free DMEM supplemented with NEAA) with or without ghrelin (100 nM) and incubated for different time (see Results). Supernatants were collected, and luciferase activity was analysed using BioLux^®^ Assay Kit on Victor 2 plate reader (PerkinElmer, Waltham, MA).

#### SNAP tag expression and SNAP-Cell^®^ 647-SiR staining

Cells transfected with pcDNA3.4 plasmid encoding SNAP tag (19.4 kDa) were grown for up to 24 h in complete DMEM and then for 12 h in low serum DMEM.

In the plate protocol, activity of SNAP protein produced by the start of cell treatment with ghrelin was irreversibly inhibited with SNAP-Cell^®^ Block (10 µM for 15 min, except for the positive control). After 2 quick washes, cells were incubated for 15–240 min in low serum DMEM (or serum-free DMEM supplemented with NEAA) with or without ghrelin (100 nM). After quick wash with PBS supplemented with 100 µg/ml CHX, cells were lysed in polysome lysis buffer (PLB) containing 20 mM Tris–HCl (pH 7.5), 250 mM NaCl, 1.5 mM MgCl2, 1 mM DTT, 0.5% Triton X-100 and freshly added 100 µg/ml CHX. Protein concentrations were measured using Bradford assay. Lysates were incubated with SNAP-Cell^®^ 647-SiR dye (2 µM) for 30–60 min at 37 °C. Equal amounts of proteins from each sample were separated by gradient polyacrylamide gel electrophoresis. Fragments of gels containing fast running unbound dye (MW = 724.9) were cut off prior to imaging. Fluorescence of de novo produced SNAP protein labelled with SNAP-Cell^®^ 647-SiR was analysed on a Typhoon Trio Imager (GE Healthcare, Chicago, Ill) using Cy5 settings.

In the glass bottom dish protocol, which was used as a control of the efficiency of cell transfection and labelling, cells were incubated with SNAP-Cell® 647-SiR (2 µM, 30 min). After removal of non-incorporated dye, samples were analysed using confocal fluorescence microscopy. Here and in (a) for Fast-FT, signal intensities in 20–25 individual cells were normalised to the mean intensity value (1 a.u.).

#### Protein synthesis analysis using Click-iT^®^ HPG Alexa Fluor 594 kit

Metabolic labelling of newly synthesised peptides was conducted using the approach described in [37]. Cells were seeded at 1.5 × 10^4^ cells/cm^2^ on MatTek glass bottom dishes and grown for 30 h in complete DMEM. Medium was replaced with serum-free DMEM supplemented with NEAA (no antibiotics) and after an overnight incubation with l-methionine-free DMEM (no NEAA and antibiotics) to deplete cellular methionine. Then, cell staining was conducted according to the kit protocol. Briefly, all components were prepared based on 200 µl of media or other solutions per one dish. First, cells were incubated for 30 min in methionine-free medium containing homopropargylglycine (HPG, an amino acid analogue of methionine with an alkyne moiety, 50 µM), with or without ghrelin (100 nM); for negative control, translation was inhibited by cycloheximide (100 µg/ml), which was added simultaneously with HPG in ghrelin (–) sample. Then, cells were washed once with pre-warmed Dulbecco-modified PBS with Ca^2+^ and Mg^2+^ (1 min), fixed with 4% PFA in PBS (12 min), washed once with PBS and twice with PBS containing 3% BSA and permeabilised with 0.5% Triton^®^ X-100 in PBS (20 min). Next, cells were washed twice with PBS/3% BSA and incubated with Click-iT^®^ reaction cocktail that contained 1 × HPG reaction buffer, CuSO_4_, Alexa Fluor^®^ 594 azide and HPG buffer additive (30 min); note that cells were protected from light starting from this step until the microscopy analysis. Cells were washed once with Click-iT^®^ reaction rinse buffer and once with PBS, which was followed by chromatin staining with NuclearMask™ Blue Stain solution in PBS (30 min). Cells were washed twice with PBS; PBS volume was adjusted to 2 ml, and then, fluorescence signals were analysed on a confocal microscope.

### Western blotting analysis

Protein isolation and Western blotting analysis were performed as described [38], except for lysis buffer composition. In this study, cells were lysed with a standard RIPA buffer (Pierce recipe) supplemented with protease and phosphatase inhibitors for 20 min on ice in a cold room. Protein samples were collected in 1.5 ml Eppendorf tubes, sonicated and clarified for 15 min at 14,000*g* and 4 °C. Protein concentrations were measured (BCA assay) and equalised. Proteins (typically 30–50 µg per lane) were separated by gradient polyacrylamide gel electrophoresis (4–20%), transferred to Immobilon membranes and immunoblotted. Blocking (1 h, room temperature) and incubation with primary antibodies (overnight, 4 °C) were performed in 5% BSA prepared in tris-buffered saline containing 0.09% Tween 20 (TBST). HRP-conjugated secondary antibodies prepared in 5% non-fat milk/TBST were applied for 1–2 h at room temperature. Blots were analysed using the LAS-3000 Imager (FujiFilm, Japan) and Image Reader LAS-3000 2.2 software. Results were quantified with ImageJ program and normalised to the levels of corresponding non-phosphorylated proteins and α-tubulin. Unless otherwise stated, protein levels were related to the corresponding normalised mean values in mock-treated cells (1 a.u.).

### Immunofluorescence analysis

For immunofluorescence, cells were seeded and grown as above on glass bottom dishes. After the treatment 100 nM ghrelin or mock for 30 min), cells were washed with Dulbecco modified PBS (supplemented with Ca^2+^ and Mg^2+^), fixed with 4% PFA for 10 min and permeabilised with 0.25% Triton X100 for 15 min. Immunostaining was performed as described [39], using antibodies against rpS6 (S235/S236), diluted 1:500 in blocking solution (5% BSA in TBST), Alexa Fluor 555-conjugated fluorescent secondary antibodies, diluted 1:1000 in blocking solution and DAPI nuclear stain. After washes in TBST and PBS, cells were left in PBS for confocal imaging. Images were analysed on an Olympus FV1000 confocal microscope (see below) with the settings recommended for DAPI and Alexa555.

### RPPA analysis

Reverse Phase Protein Array analysis [40] using over 300 antibodies was conducted by Functional Proteomics Group of MD Anderson Cancer Centre (University of Texas, USA). Note that p-eEF2 (Thr56) and p-eEF2K levels could not be examined by RPPA at the time of analysis. Samples (1 mg/ml proteins) were prepared as for Western blotting analysis in a dye-free Laemmle buffer and shipped for the analysis on dry ice.

### Polysome profiling

Polysomes were prepared as described [24]. Briefly, cells were grown on 15 cm^2^ Petri dishes, incubated in low serum DMEM for 6 h, treated with ghrelin (100 nM) as described in Results and quickly washed 1 × with ice-cold PBS containing 100 µg/ml CHX. Immediately, 0.5 ml of PLB was applied (with 15 mM MgCl_2_, instead of 1.5 mM MgCl_2_), and cells were collected in 1.5 Eppendorf tubes. After 10 min incubation on ice, lysates were clarified by centrifugation for 10 min at 16,000*g*. Protein concentrations in supernatants containing ribosomes were measured (Bradford assay) and equalised. Samples were fractionated using centrifugation at 210,000*g* in 10–60% sucrose gradients. Differences in ribosome density along mRNA molecules were evaluated by calculating the ratio between monosome and polysome fractions (the area under a curve).

### Cap (m^7^GTP) pull-down assay

Assay was performed as described [41] with modifications. HEK293^GHS-R1α-EGFP+^ cells were grown on 15 cm Petri dishes, incubated in low serum DMEM (or serum-free DMEM supplemented with NEAA) for 6 h and treated as shown in Fig. 2. Cells were then washed with PBS and lysed in 1 ml of buffer A (50 mM Tris–HCl, pH 7.0, 100 mM NaCl, 1 mM EDTA, 0.5% NP-40 supplemented with protease and phosphatase inhibitors) on ice for 20 min. Lysates were cleared by centrifugation (16,000*g* for 10 min at 4 °C). m^7^GTP-agarose beads (∼50 μl of 50% slurry per sample) were equilibrated in buffer B (15 mM Tris–HCl, pH7.2, 100 mM NaCl, 1 mM EDTA, 0.1% NP-40). Equal volumes of supernatants (∼ 1.2 ml) were incubated with m^7^GTP-agarose beads for 2 h at 4 °C on an end-over-end rotor. The beads were collected by centrifugation (500*g* for 5 min at 4 °C) and washed four times with 1 ml of buffer B. Bound proteins were eluted with 1 × SDS loading buffer (20 min at 40 °C) and analysed by Western blotting along with the inputs. Independent experiments were performed in duplicate.

### Confocal microscopy analysis of GFP-GHS-R1α internalisation, SNAP production, nascent protein synthesis and Ca^2+^ levels

Analysis of GFP-GHS-R1α internalisation, and levels of SNAP (using SNAP-Cell® 647-SiR), total nascent proteins (using Alexa Fluor 594) and Ca^2+^ (using Fura Red) was conducted at 37 °C on an inverted Olympus FV1000 confocal laser scanning microscope with controlled humidity, temperature and CO_2_. NuclearMask™ Blue Stain was excited at 405 nm (10% laser power), with emission collected at 420–460 nm. GFP was excited at 488 nm (3–5% of laser power), emission collected at 500–560 nm. Fura Red and Alexa Fluor 594 were excited at 543 nm (40% of laser power), emission collected at 560–660 nm. SNAP-Cell^®^ 647-SiR was excited at 633 nm (1.4–15% of laser power) with emission collected at 650–750 nm. Acquisition was performed using oil immersion UPLSAPO 60 × /1.35 Super Apochromat objective and UPLSAPO 20 × /0.75 objective (for Alexa Fluor 594). In all experiments, differential interference contrast (DIC) images were taken to compliment stacks of 2–10 fluorescence images, collected with 0.5 µm steps (80 µm aperture). For the live cell analysis of GFP-GHS-R1α internalisation, images were taken every 5 min. For quantitative analysis, signal intensities in whole cells (SNAP-Cell® 647-SiR, Alexa Fluor 594 and Fura Red), nuclei (NuclearMask™), as well as in cytosol and the plasma membrane areas (GFP-GHS-R1α), were averaged separately for > 20 cells. Analysis was performed using FV1000 Viewer software (Olympus) and Microsoft Excel.

Fluorescence lifetime imaging microscopy (FLIM) of Ca^2+^ levels using OGB-1 dye was performed at 37 °C on an upright laser scanning Axio Examiner Z1 (Carl Zeiss) microscope equipped with 20 × /1.0 W-Plan Apochromat dipping objective, integrated T and Z-axis control, using ps supercontinuum SC400-4 (Fianium, UK) laser, DCS-120 confocal time-correlated single photon counting (TCSPC) scanner, photon counting detector and SPCM software (Becker & Hickl GmbH, Berlin, Germany). OGB-1 was excited using 488 nm laser, with emission collected at 512–536 nm. Data were analysed with SPCImage (B&H) and Excel software. Fluorescence lifetime (LT) of OGB-1 was calculated using monoexponential decay curves. Frequency histograms demonstrating the distribution of OGB-1 LT were obtained for three individual focal planes within each field of view (256 × 256-pixel matrixes). Cumulative LT distribution histograms were produced according to the algorithm developed in [42].

Here and elsewhere figures were assembled using Adobe Photoshop and Illustrator software.

### Reverse transcription and qPCR analysis

HEK293^GHS-R1α-EGFP^ cells transfected with plasmids encoding *G*Luc and SNAP reporters were seeded at 5 × 10^5^ cells per well on 6-well plates, grown sequentially in complete DMEM (24 h) and in DMEM supplemented with 1% FBS (20 h) and treated with mock or 100 nM ghrelin for 30 min. RNA was isolated immediately using RNeasy® Plus Universal Mini Kit in two replicates for each condition (the content of 3 wells was combined in each case, giving approximately 3–4 × 10^6^ cells for a replicate). Reverse transcription was performed using 1.5 µg of total RNA and High-Capacity cDNA Reverse Transcription Kit on a MiniAmp Plus Thermal Cycler (Applied Biosystems). qPCR was conducted on the LightCycler 480 II (Roche) using the SensiFAST SYBR Lo-ROX kit and primers to SNAP (F: 5´-GAAATGAAGCGCACCACCC and R: 5´-GGTGAAAGTAGGCGTTGAGC), *G*Luc (F: 5´-AAGTTCATCCCAGGACGCTG and R: 5´-GTCAGAACACTGCACGTTGG) and β-actin (F: 5´-CGGCTACAGCTTCACCACCACG and R: 5´-AGGCTGGAAGAGTGCCTCAGGG). All samples were measured in triplicate; SNAP and *G*Luc expression was normalised to β-actin.

### Total cellular ATP analysis

Total cellular ATP levels in HEK293^GHS-R1α-EGFP^ cells incubated in the medium containing glucose and galactose/FCCP (energy stress conditions) were measured using CellTiter-Glo® assay, following the manufacturers’ protocol. Briefly, cells treated in 100 µl of DMEM as indicated in Results section were lysed with 100 µl of CellTiterGlo^®^ reagent. After intensive shaking (2 min, 37 °C), the samples (200 µl) were transferred into wells of white 96-well plates (Greiner Bio One) and analysed on a Victor 2 plate reader under standard luminescence settings.

### Statistical analysis

Data were analysed with two-sided independent *T* test or one-way ANOVA followed by Dunnett or Tukey post hoc tests. Results of at least 3 independent experiments were used (unless otherwise stated). *p* values of ≤ 0.05 were deemed as statistically significant. Plate reader-based experiments were conducted using 3–6 technical replicates for each experimental condition. Graphically, the results are presented as mean ± SD, boxes and whiskers (with median, IQR and either min/max values or 5% and 95% values) or/and as individual data points.

## Results

### Activation of ghrelin signalling increases mRNA translation rate

**Fig. 1:**
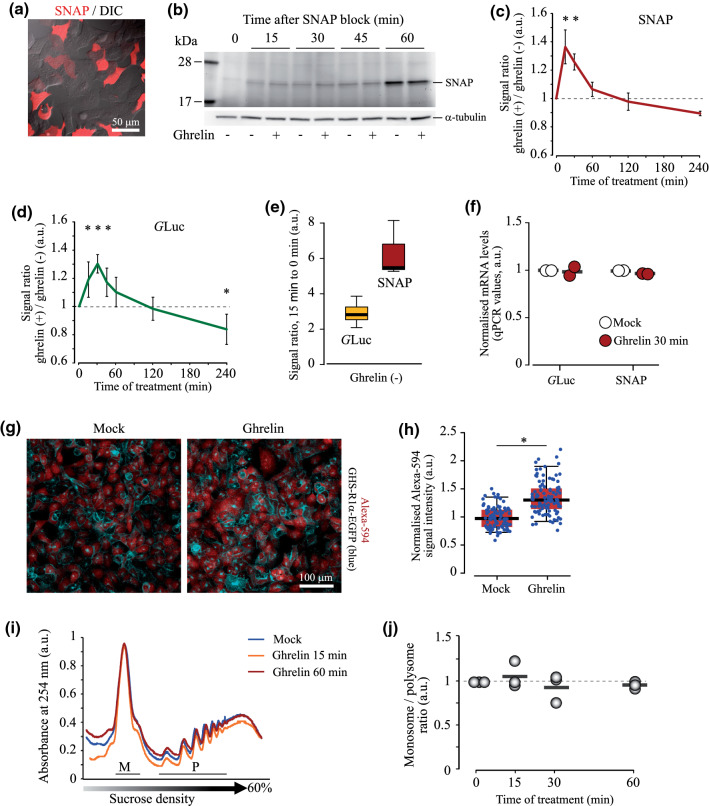
Changes in protein synthesis in HEK293^GHS-R1α-EGFP+^ cells in response to GHS-R1α activation by ghrelin. **a** Efficiency of cell transfection with a plasmid encoding SNAP (> 55% SNAP-positive cells 24 h after the onset of transfection). **b** Representative fluorescent image of the gel showing the amounts of SNAP protein (647-SiR fluorescence) produced in cells treated with 100 nM ghrelin for 0–60 min. **c** The ratio of SNAP production in cells treated with ghrelin or mock (medium) for 0–240 min, corresponding to (**b**). **d** Dynamics of *G*Luc production by cells treated with 100 nM ghrelin. The ratio of *G*Luc signals in the supernatants of cells treated with ghrelin or mock (medium) for indicated time. Note that over 90% of the newly produced *G*Luc is rapidly secreted from cells, thus minimising risks of intracellular protein degradation. **e** Signal-to-noise ratios calculated for the 15-min time point vs 0 time point (no ghrelin) of *G*Luc- and SNAP-based analysis. **f** RT-qPCR analysis of SNAP and *G*Luc mRNA levels in cells transfected with the corresponding plasmids and treated with mock or 100 nM ghrelin for 30 min (*N* = 2). **g** Representative images (stacks of 7 focal planes) of Alexa Fluor 594 and EGFP fluorescence (showing nascent peptides (in red) and GHS-R1α (in blue), respectively) in mock and ghrelin-treated cells (100 nM ghrelin for 30 min); Click-iT^®^ HPG Alexa Fluor 594 Protein Synthesis Assay Kit was used. **h** Quantitative analysis of the Alexa Fluor 594 intensity signals in ghrelin- and mock-treated cells. **i**, **j** Distribution of ribosomes in sucrose density gradients and monosome/polysome (M/P) ratio in HEK293^GHS-R1α-EGFP+^ cells treated with 100 nM ghrelin for 0–60 min (*N* = 3). Error bars in (**c**, **d**) are presented for experiments repeated at least three times. In (**e**, **h**), boxes show median values and the interquartile range; in (**e**), whiskers extend to the minimal and maximal values; in (**h**), Altman-style whiskers extend to 5th and 95th percentiles, and all analysed cells are represented by data points (*n* > 90 in each group, *N* = 3 independent experiments). Data are shown as individual data points (circles in **f** and **j**; dots in **h**), mean values (horizontal bars, **f, h** and **j**) and mean ± SD. Asterisks show significant difference between ghrelin- and mock-treated (0-time point) cells. Statistical details: **b, c** Effect of ghrelin treatment time: SNAP (5, 15) = 27.048, *p* < 0.001, one-way ANOVA, *p* < 0.001 for 15 and 30 min, *p* = 0.145 for 45 min, *p* = 0.597 for 60 min, *p* = 0.991 for 120 min, *p* = 0.193 for 240 min, Dunnett post hoc test vs mock (0 time point) treatment (*N* = 4 for 0, 15, 30 min; *N* = 3 for 45, 60, 120, 240 min). **d** Effect of ghrelin treatment time: *G*Luc (6,28) = 14.183, *p* < 0.001, one-way ANOVA, *p* = 0.015 for 15 min, *p* < 0.001 for 30 min, *p* = 0.032 for 45 min, *p* = 0.341 for 60 min, *p* = 0.999 for 120 min, *p* = 0.043 for 240 min, Dunnett post hoc test vs mock (0 time point) treatment (*N* = 5). **h** Effect of ghrelin treatment, *N* = 3, independent samples *T* test, Alexa Fluor 594 *t*(4) = – 3.12, *p* = 0.036

We first examined whether ghrelin treatment causes detectable changes in de novo protein production in HEK293^GHS-R1α-EGFP+^ cells. To address this, we analysed the rate of protein production using a Click-iT® HPG Alexa Fluor 594 Kit for metabolic labelling, two fluorescent reporters (Fast-FT and SNAP-tag/SNAP-Cell® 647-SiR) and one bioluminescent reporter (*Gaussia* luciferase, *G*Luc). All reporters were effectively delivered into the cells (as exemplified in Fig. 1a with SNAP staining).

The nascent Fast-FT rapidly (< 1 h) maturates into a blue-emitting fluorescent protein, which is then slowly (> 20 h) converted into a red emitter [35]. We found no significant difference in blue emission and blue/red signal ratio between ghrelin- and mock-treated cells over a 4-h time frame (not shown). However, the Fast-FT is a relatively slow-responding reporter, and if ghrelin induced transient and/or moderate changes in protein synthesis, the maturation rate of Fast-FT would not have allowed detecting such changes. Thus, we conducted similar experiments with faster responding SNAP and *G*Luc assays. Indeed, we observed a rapid transient elevation in both reporters’ signal upon ghrelin stimulation: protein production increased by approximately 30% and reached its maximum within 15–30 min of ghrelin treatment (Figs. 1b–e, S1a–c). SNAP reporter responded quicker than *G*Luc (Figs. 1c, d, S1c). After 1.5–2 h of ghrelin treatment, the rate of protein production progressively decreased (Fig. 1c, d).

A rapid increase in protein production, seen under ghrelin treatment in our cell model, strongly suggests activation of mRNA translation rather than gene transcription. Indeed, the levels of SNAP and *G*Luc mRNA did not change after 30 min treatment with ghrelin (Fig. 1f). Furthermore, confocal microscopy analysis of HPG/Alexa Fluor 594 incorporation in newly produced proteins showed an approximately 30% increase in mRNA translation in HEK293^GHS-R1α-EGFP+^ cells treated with ghrelin for 30 min (Figs. 1g, h, S1d). Therefore, next, using polysome profiling method we analysed changes in the ribosome occupancy of mRNA molecules induced by ghrelin. This analysis is based on differential distribution of polysomes in sucrose gradients, which allows separating monosome and polysome fractions. An enhanced translation rate is typically associated with a decrease in monosome/polysome ratio; however, we failed to find changes in this ratio within 60 min upon ghrelin addition (Fig. 1i, j).

This result, at a first glance counterintuitive, has at least two possible explanations: (1) polysome profiling assay is not sensitive enough to detect a 30% increase in translation of the reporter constructs; (2) ghrelin-mediated changes in initiation and elongation rates compensate for each other. Indeed, elevated initiation rate is expected to boost ribosome occupancy of mRNA; but increased elongation rate might reduce the number of ribosomes per mRNA molecule because ribosomes accomplish protein synthesis and dissociate from mRNA faster [43].

### Activation of ghrelin signalling has marginal effects on translation initiation

**Fig. 2:**
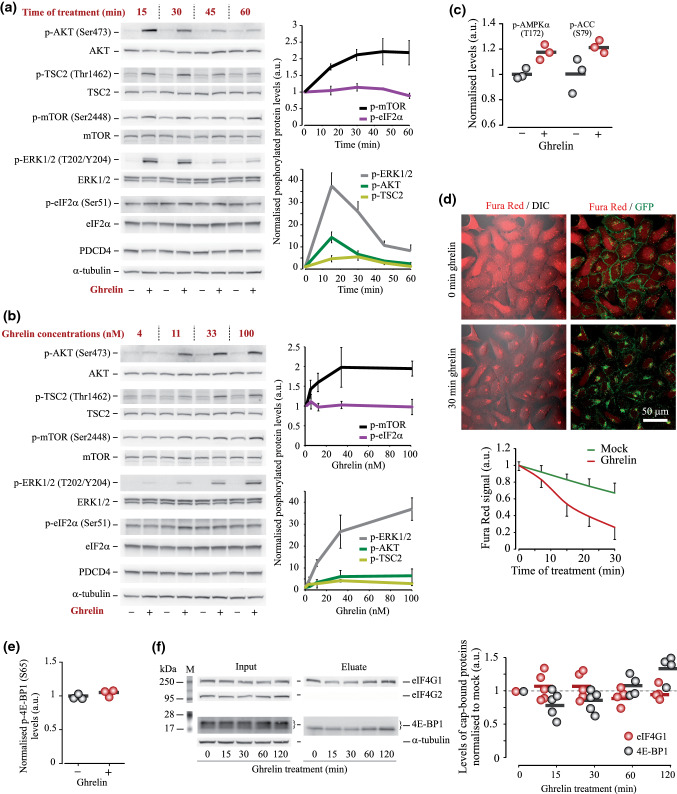
Effects of GHS-R1α activation by ghrelin on the pathways regulating translation initiation and on the translation initiation complex in HEK293^GHS-R1α-EGFP+^ cells. **a**,** b** Western blotting analysis of time- (**a**) and concentration- (**b**) dependent effects of ghrelin on the activity of AKT/mTOR and ERK signalling cascades, eIF2 phosphorylation and PDCD4 levels. In (**a**), 100 nM ghrelin was used; in (**b**), cells were treated for 30 min. **c** RPPA analysis of the effects of ghrelin treatment (500 nM, 1 h) on AMPK activity, reported by p-AMPKα (T172) and p-ACC (S79) levels (*N* = 3). **d** Effect of ghrelin on GHS-R1α internalisation and cytosolic Ca^2+^ levels: live cell confocal imaging analysis of translocation of GFP-tagged ghrelin receptor to cytosol and changes in Fura Red fluorescence upon ghrelin treatment. Note that a large proportion of GHS-R1α undergoes internalisation and aggregates in non-treated cells due to the constitutive activity of the receptor. Fura Red signals decrease upon Ca^2+^ elevation; photo-bleaching of the dye is shown as mock. In all experiments (*N* = 3), cells were pre-incubated in DMEM supplemented with 1% FBS for 12–14 h and then treated with 100 nM ghrelin. Fluorescence images are stacks of three (GFP) and five confocal planes (Fura Red) taken with 0.5 µm steps. **e** RPPA analysis of the effect of ghrelin treatment (500 nM, 1 h) on p-4E-BP1 (S65) levels; phosphorylation blocks the capacity of 4E-BP1 to inhibit initiation. **f** Composition of cap-bound proteins-regulators of translation initiation; changes in eIF4G and 4E-BP1 protein levels during 2 h treatment with ghrelin are shown. Input protein analysis is used as control; α-tubulin and eIF4G2 are detected only in the input samples, demonstrating specificity of cap binding. Data are shown as individual data points (gradient circles), mean values (horizontal bars) and mean ± SD. Asterisks have not been used because of the complexity of plots; see statistical significance report below. Statistical details: **a** effect of ghrelin treatment time: (a) p-AKT (4,8) = 39.625, *p* < 0.001, one-way ANOVA, *p* = 0.011 for 15 min, *p* = 0.024 for 30 min, *p* = 0.005 for 45 min, *p* = 0.068 for 60 min, Dunnett post hoc test vs mock treatment (*N* = 3); (b) p-TSC2 (4,8) = 12.738, *p* = 0.002, one-way ANOVA, *p* = 0.046 for 15 min, *p* = 0.038 for 30 min, *p* = 0.057 for 45 min, *p* = 0.198 for 60 min, Dunnett post hoc test vs mock treatment (*N* = 3); (c) p-mTOR (4,12) = 14.25, *p* < 0.001, one-way ANOVA, *p* < 0.001 for 15 and 30 min, *p* = 0.008 for 45 min, *p* = 0.007 for 60 min, Dunnett post hoc test vs mock treatment (*N* = 4); (d) p-ERK (4,20) = 134.963, *p* < 0.001, one-way ANOVA, *p* < 0.001 for 15, 30 and 45 min, *p* = 0.001 for 60 min, Dunnett post hoc test vs mock treatment (*N* = 6); (e) p-eIF2a (4,8) = 3.162, *p* = 0.078, one-way ANOVA, *p* = 0.614 for 15 min, *p* = 0.164 for 30 min, *p* = 0.035 for 45 min, *p* = 0.132 for 60 min, Dunnett post hoc test vs mock treatment (*N* = 3). **b** Effect of ghrelin concentration: (a) p-AKT (4,8) = 4.92, *p* = 0.019, one-way ANOVA, *p* = 0.995 for 3 nM, *p* = 0.421 for 11 nM, *p* = 0.033 for 33 nM, *p* = 0.022 for 100 nM, Dunnett post hoc test vs mock treatment (*N* = 3); (b) p-TSC2 (4,8) = 2.872, *p* = 0.08, one-way ANOVA, *p* = 0.424 for 3 nM, *p* = 0.26 for 11 nM, *p* = 0.023 for 33 nM, *p* = 0.246 for 100 nM, Dunnett post hoc test vs mock treatment (*N* = 3); (c) p-mTOR (4,12) = 8.382, *p* < 0.001, one-way ANOVA, *p* = 0.214 for 3 nM, *p* = 0.025 for 11 nM, *p* < 0.001 for 33 and 100 nM, Dunnett post hoc test vs mock treatment (*N* = 4); (d) p-ERK (4,20) = 80.687, *p* < 0.001, one-way ANOVA, *p* = 0.311 for 3 nM, *p* < 0.001 for 11 nM, 33 nM and 100 nM, Dunnett post hoc test vs mock treatment (*N* = 6); e) p-eIF2a (4,8) = 0.523, *p* = 0.722, one-way ANOVA, *p* = 0.77, 0.989, 1 and 0.993 for 3 nM, 11 nM, 33 nM and 100 nM, accordingly, Dunnett post hoc test vs mock treatment (*N* = 3). **c** RPPA analysis, *N* = 3, independent samples T test for: a) AMPK (pT172): t(4) = – 2.507, *p* = 0.066; b) ACC (pS79): *t*(4) = – 2.053, *p* = 0.109. **d** Confocal analysis of Fura Red signal (30 min after ghrelin treatment), independent samples *T* test, (*N* = 3), T(24) = – 26.197, *p* < 0.001. **f** Effect of ghrelin treatment on the cap-binding capacity of eIF4G1 and 4E-BP1: (a) eIF4G1 (4,16) = 0.391, *p* = 0.812, one-way ANOVA for the effect of treatment time, *p* = 0.863, 0.916, 1 and 0.753 for 15, 30, 60 and 124E-BP1 eIF4G (4,16) = 5.456, *p* = 0.006, one-way ANOVA for the effect of treatment time, *p* = 0.271, 0.482, 0.939 and 0.055 for 15, 30, 60 and 120 min, respectively, Dunnett post hoc test vs mock (0 time point) treatment (*N* = 5 for 15 and 30 min and *N* = 4 for 60 and 120 min of ghrelin treatment)

Translation initiation is thought to be the main rate-limiting step in protein synthesis [44], though the efficiency of initiation in many ways depends on elongation [45]. Bearing this in mind, we examined the effects of ghrelin on several parameters characterising initiation efficiency. The results of analysis of generic signalling pathways involved in the regulation of translation initiation were not very conclusive. On one hand, Western blotting analyses demonstrated time- and concentration-dependent activation of AKT/mTOR and ERK1/2 signalling cascades known to up-regulate translation initiation (Fig. 2a, b, see also Fig. S2). Phosphorylation of eIF2a at Ser51, which is known to inhibit total protein production, was not affected by ghrelin treatment in a broad range of ghrelin concentrations (4–100 nM) and time (15–60 min) (Fig. 2a, b). At the same time, AMPK pathway, which inhibits translation, showed a trend towards activation, as reported by increased p-AMPKα (T172) and p-ACC (S79) levels (Fig. 2c, RPPA data). In agreement with AMPK data, further analysis using live cell confocal microscopy revealed an elevation of cellular Ca^2+^ levels concomitant with increased GHS-R1α internalisation in response to ghrelin treatment (Fig. 2d, Fig. S3).

Next, using Western blotting and RPPA, we analysed the levels of PDCD4 and 4E-BP1 proteins, known to inhibit cap-dependent translation initiation [46, 47], and found no effects of ghrelin treatment (Figs. 2a, b, e, S2). To examine whether ghrelin-mediated signalling affects the activity of eIF4F complex, which is essential for cap-dependent initiation, we carried out m^7^GTP pull down assay. Typically, recruitment of the scanning 40S ribosome subunit to the 5' cap of mRNAs depends on the ratio between eIF4G and 4E-BP, which both interact with eIF4E (the ‘core’ component of eIF4F complex) and can be immobilised on and eluted from m^7^GTP resin. Non-phosphorylated 4E-BP acts as initiation repressor by disrupting interaction between eIF4E and eIF4G. The assay revealed negligible effects of ghrelin on the repertoire of cap-bound proteins (Fig. 2f). 4E-BP1 levels showed a negative trend during the first 30 min of the treatment and a positive trend within a 60–120 min time frame. Taken together, these results suggest that in our model ghrelin does not exert major short-term effects on eIF4F complex, which directly regulates translation initiation.

### Ghrelin specifically decreases phosphorylation of eEF2

**Fig. 3:**
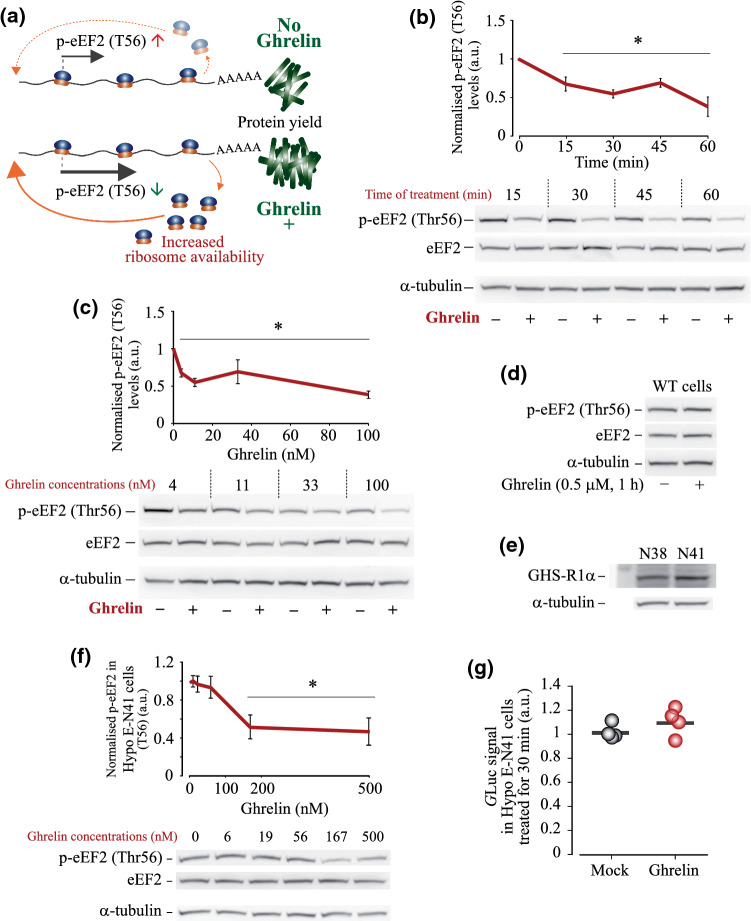
Effects of GHS-R1α activation by ghrelin on eEF2 phosphorylation. **a** Proposed contribution of elongation into ribosome turnover and protein production de novo. Decreased eEF2 phosphorylation is expected to increase elongation rate, ribosome availability and protein production levels. **b**, **c** Western blotting analysis of time- and concentration-dependent changes in eEF2 (Thr56) phosphorylation induced in HEK293^GHS-R1α-EGFP+^ cells by ghrelin treatment; **b** 100 nM ghrelin for 15–60 min and **c** 4–100 nM ghrelin for 30 min. **d** Analysis of eEF2 phosphorylation in parental HEK293 cells lacking GHS-R1α. **e** Analysis of GHS-R1α protein expression in mouse embryonic hypothalamus cell lines E-N38 and E-N41; in E-N41, GHS-R1α protein levels are ~ 60% higher. **f** Ghrelin concentration-dependent decrease in p-eEF2 (Thr56) levels in E-N41 cells (30 min treatment). **g** Changes in the extracellular *G*Luc signals triggered by ghrelin treatment in E-N41 cells (200 nM, 30 min). Cells were pre-incubated for 14–16 h in serum-free DMEM supplemented with NEAA. Data are shown as individual data points (gradient circles), mean values (horizontal bars) and mean ± SD. Asterisks indicate significant difference between non-treated (0 time point or 0 ghrelin concentration) and ghrelin-treated cells. Statistical details: **b** effect of ghrelin treatment time: p-eEF2 (4,20) = 124.011, *p* < 0.001, one-way ANOVA, *p* < 0.001 for 15, 30, 45 and 60 min, Dunnett post hoc test vs mock treatment (*N* = 6). **c** Effect of ghrelin concentration: p-eEF2 (4,20) = 45.25, *p* < 0.001, one-way ANOVA, *p* < 0.001 for 3 nM, 11 nM, 33 nM and 100 nM, Dunnett post hoc test vs mock treatment (*N* = 6). **f** effect of ghrelin concentration: p-eEF2 (5,12) = 27.815, *p* < 0.001, one-way ANOVA, *p* < 0.001 for 167 nM and 500 nM ghrelin, Dunnett post hoc test vs mock treatment (*N* = 3). **g** effect of ghrelin treatment, *N* = 4, independent samples *T* test: *G*Luc *t*(6) = – 1.234, *p* = 0.181

Since activation of GHS-R1α had little effect on translation initiation, we hypothesised that elevated translation elongation rate contributes to an increase in protein production in response to ghrelin (Fig. 3a). To explore this, we examined the phosphorylation of eEF2 at Thr56, which inhibits eEF2 activity and decreases elongation rate. Western blotting analysis revealed pronounced decrease in p-eEF2 (Thr56) levels upon stimulation of HEK293^GHS-R1α-EGFP+^ cells with ghrelin in a broad range of concentrations and treatment time (Figs. 3b, c, S4a, b). These changes were consistent with well-characterised activation of PI3K/AKT/GSK-3β, PI3K/AKT/mTOR and Raf-1/MEK/ERK1/2 signalling pathways [10, 11, 48], which was also observed in our model. A decrease in p-eEF2 levels was seen in cells treated with as low as 4 × 10^–9^ M ghrelin (Fig. 3c). The response of eEF2 to ghrelin sustained over the 15–60 min time window (Fig. 3b). Phospho-eEF2 levels decreased in GHS-R1α-specific manner since the response was absent in the parental HEK cells (Fig. 3d). Next, we examined whether ghrelin could affect eEF2 phosphorylation in cells expressing GHS-R1α endogenously. For this, we have chosen embryonic mouse hypothalamus cells E-N38 and E-N41 with detectable GHS-R1α protein levels (Fig. 3e). The E-N41 cell line showed ~ 60% higher levels of GHS-R1α expression; hence, it was used for the analysis of eEF2 phosphorylation. Although the response of E-N41 cells was weaker than that of HEK293^GHS-R1α-EGFP+^ cells, we observed a decrease in p-eEF2 (Thr56) levels at > 150 nM ghrelin concentrations (Fig. 3f). Furthermore, a positive trend in *G*Luc protein synthesis was detected in E-N41 cells treated with 200 nM ghrelin for 30 min (Fig. 3g).

### Phosphorylation of eEF2K at Ser366 drives the ghrelin-dependent decrease in p-eEF2 levels

**Fig. 4:**
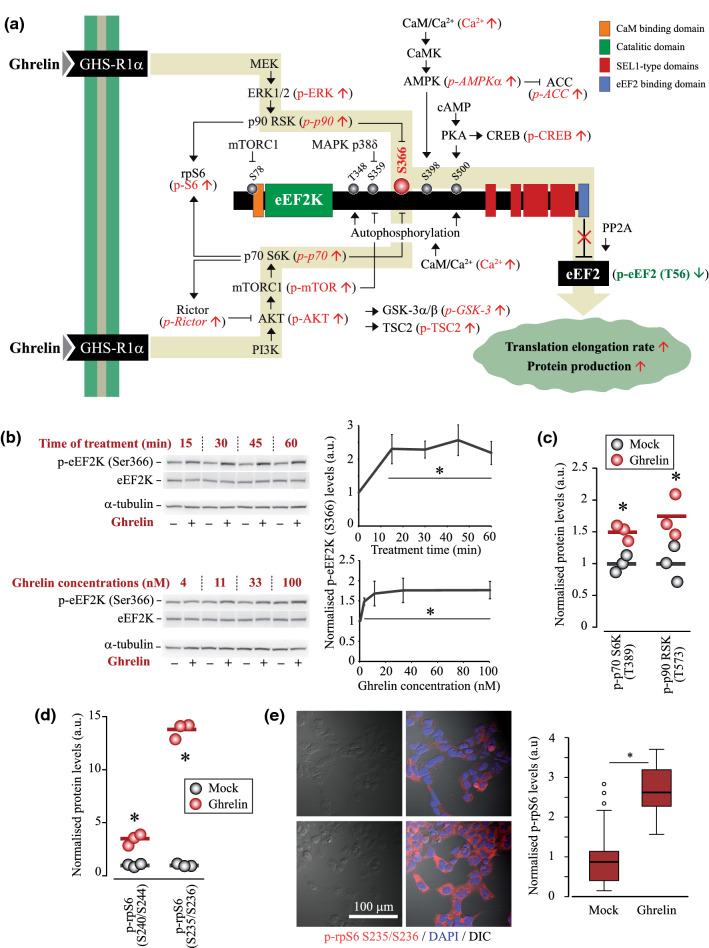
Proposed mechanism of eEF2K regulation upon GHS-R1α activation and analysis of pathways inhibiting eEF2K activity through Ser366 phosphorylation. **a** Schematics of eEF2K posttranslational modifications and ghrelin-triggered cascades modulating eEF2K activity and eEF2 phosphorylation state. Measured in this study parameters (using confocal microscopy, Western blotting and/or RPPA analysis of HEK293^GHS-R1α-EGFP+^ cells) are in brackets, shown in red or green; italic is used for *RPPA only* data. Except for p-AMPKα (T172) and p-ACC (S79), which both demonstrated positive trends, and CREB (S133), which was shown in one replicate, all changes are statistically significant (one-way ANOVA and independent samples T test). The proposed pathways that de-suppress eEF2 upon ghrelin treatment are highlighted by a background yellow-grey arrow. **b** Time- and concentration-dependent effects of ghrelin treatment on p-eEF2K (S366) levels. **c**, **d** RPPA analysis of p-p70S6K (T389), p-p90RSK (T573), p-rpS6 (S240/S244) and p-rpS6 (S235/S236) levels. **e** Confocal microscopy analysis of p-rpS6 (S235/S236) levels; stacks of 6 fluorescence images taken with 0.5 µm step and single-plane DIC images show cellular p-rpS6 levels (Alexa Fluor 555) and nuclei (DAPI); 20 cells were analysed in three mock- and ghrelin-treated samples. Cells were pre-incubated for 15–16 h in serum-free DMEM supplemented with NEAA. In RPPA experiments, cells were treated with 500 nM ghrelin for 1 h, in confocal imaging experiments—with 100 nM ghrelin for 30 min. Data are shown as individual data points (gradient circles), mean values (horizontal bars) and mean ± SD. Asterisks indicate significant difference between non-treated (0 time point or 0 ghrelin concentration) and ghrelin-treated cells. Statistical details: **b** effect of ghrelin on p-eEF2K (S366): upper panel, effect of treatment time (4,20) = 16.730, *p* < 0.001, one-way ANOVA, *p* < 0.001 for 15 and 60 min, *p* = 0.003 for 30 min, *p* = 0.002 for 45 min, Dunnett post hoc test vs mock treatment (N = 6); lower panel, effect of ghrelin concentration (4,20) = 12.677, *p* < 0.001, one-way ANOVA; *p* = 0.003 for 3 nM, *p* < 0.001 for 11 nM, 33 nM and 100 nM, Dunnett post hoc test vs mock treatment (*N* = 6). (**c**) RPPA analysis, *N* = 3, independent samples *T* test; p70S6K(pT389): *t*(4) = – 4.707, *p* = 0.009; p90RSK(pT573): *t*(4) = – 2.892, *p* = 0.044. **d** RPPA analysis, *N* = 3, independent samples *T* test; rpS6(pS240/S244): *t*(4) = – 7.535, *p* = 0.002; rpS6(pS235/S236): t(4) = – 29.138, *p* < 0.001; GSK-3α/β (pS21/pS9, not shown graphically): *t*(4) = – 7.29, *p* = 0.002; Rictor (pT1135, not shown graphically): *t*(4) = – 3.69141, *p* = 0.021. **e** Immunostaining analysis, *N* = 3, independent samples *T* test. *t*(51) = – 8.739, *p* < 0.001

Next, we sought to identify the mechanisms underlying the effects of ghrelin on the activity of eEF2. The levels of eEF2 phosphorylation depend on the activities of its kinase eEF2K and its phosphatase PP2A (Fig. 4a). The regulation of eEF2K activity is quite complex, and its key components are depicted in Fig. 4a. Briefly, the activity of eEF2K can be increased via phosphorylation at Thr348/Ser500 (Ca^2+^ and CaM-dependent autophosphorylation) and S398 (AMPK pathway). In turn, phosphorylation at Ser78/Ser359 (mTOR and p38δ MAPK) and Ser366 site (mTOR/p70 S6K and ERK/p90 RSK pathways) inhibits eEF2K. The results of Western blotting analysis showed a robust activation of ERK and mTOR pathways under ghrelin treatment (Fig. 2a, b), which suggested that phosphorylation at Ser366 position was likely to contribute to eEF2 activation (de-suppression, Fig. 4a). To test this hypothesis, we analysed changes in eEF2K phosphorylation state induced by ghrelin treatment.

**Fig. 5:**
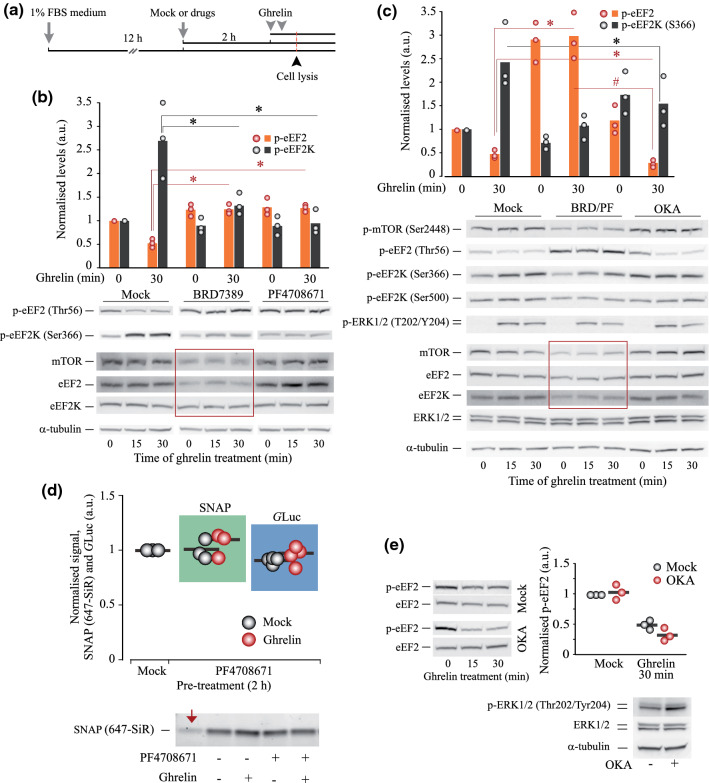
Roles of rpS6 kinases and PP2A in ghrelin-induced activation of eEF2 and protein synthesis. **a** The schematic of HEK293^GHS-R1α-EGFP+^ cells treatment. **b** Changes in eEF2 and eEF2K phosphorylation induced by ghrelin after inhibition of either p90 RSK (10 µM BRD7389) or p70 S6K (1 µM PF4708671). **c** Effects of ghrelin on p-eEF2 (Thr56) and p-eEF2K (Ser366 and Ser500) levels in cells upon inhibition of p90 RSK and p70 S6K (double-treatment with BRD7389/PF4708671) or PP2A (OKA, 50 nM). In (**b**, **c**), cells were pre-treated with kinase or phosphatase inhibitors for 2 h prior to ghrelin addition (100 nM, 30 min). Changes in p-mTOR and p-ERK levels were assessed to confirm the GHS-R1α activation. **d** Changes in SNAP and *G*Luc production in cells treated with ghrelin upon inhibition of p70 S6K (1 µM PF4708671). Cells were pre-treated for 2 h and then treated with ghrelin (100 nM, 30 min) in the presence of PF4708671; an arrow shows the residual amounts of SNAP in cells lysed immediately after their treatment with SNAP block. **e** Comparative analysis of eEF2 response to ghrelin in resting and OKA-treated cells [extracted from (**c**)]. The bottom panel shows that the pre-treatment with OKA causes an increase in phosphorylation of ERK, a common target of PP2A. Red rectangles (**b**, **c**) highlight effects of BRD7389 on total levels of mTOR, eEF2 and eEF2K proteins. Red asterisks indicate significant difference in the p-eEF2 (Thr56) response to ghrelin in mock-treated vs BRD- and PF-treated cells (**b**), or in mock-treated vs BRD/PF- and OKA-treated cells (**c**), red hash signs—significant difference in p-eEF2 response to ghrelin between BRD/PF- and OKA-treated cells. Black asterisks show the same for p-eEF2K (Ser366) levels. Columns/horizontal bars and gradient circles show mean values and individual data points, respectively. Statistical details: **b** effects of p90 RSK or p70 S6K inhibition on the ghrelin-induced changes in eEF2 and eEF2K phosphorylation; (a) p-eEF2 (2,6) = 29.442, *p* < 0.001, one-way ANOVA, *p* < 0.001 for mock vs BRD, *p* = 0.001 for mock vs PF, Dunnett post hoc test vs mock treatment (*N* = 3); (b) p-eEF2K (2,6) = 10.13, *p* = 0.012, one-way ANOVA for the effect of treatments (BRD and PF), *p* = 0.024 for mock vs BRD, *p* = 0.01 for mock vs PF, Dunnett post hoc test vs mock treatment (*N* = 3). **c** Effects of rpS6 kinase and PP2A inhibition on the ghrelin-induced changes in eEF2 and eEF2K phosphorylation; (a) p-eEF2 (2,6) = 61.086, *p* < 0.001, one-way ANOVA for the effect of treatments (BRD/PF and OKA), *p* < 0.001 for mock vs BRD/PF, *p* = 0.043 for mock vs OKA, *p* < 0.001 for BRD/PF vs OKA, Tukey post hoc test (*N* = 3); (b) p-eEF2K (2,6) = 8.378, *p* = 0.018, one-way ANOVA for the effect of treatments (BRD/PF and OKA), *p* = 0.127 for mock vs BRD/PF, *p* = 0.015 for mock vs OKA, *p* = 0.263 for BRD/PF vs OKA, Tukey post hoc test (*N* = 3). **d** Effect of p70 S6K inhibition on the changes in *SNAP* (*N* = 3) and *G*Luc (*N* = 4) translation caused by ghrelin treatment, independent samples *T* test; SNAP: Alexa Fluor 594 *t*(4) = – 0.7822, *p* = 0.478; *G*Luc: *t*(6) = – 0.676, *p* = 0.524

**Fig. 6:**
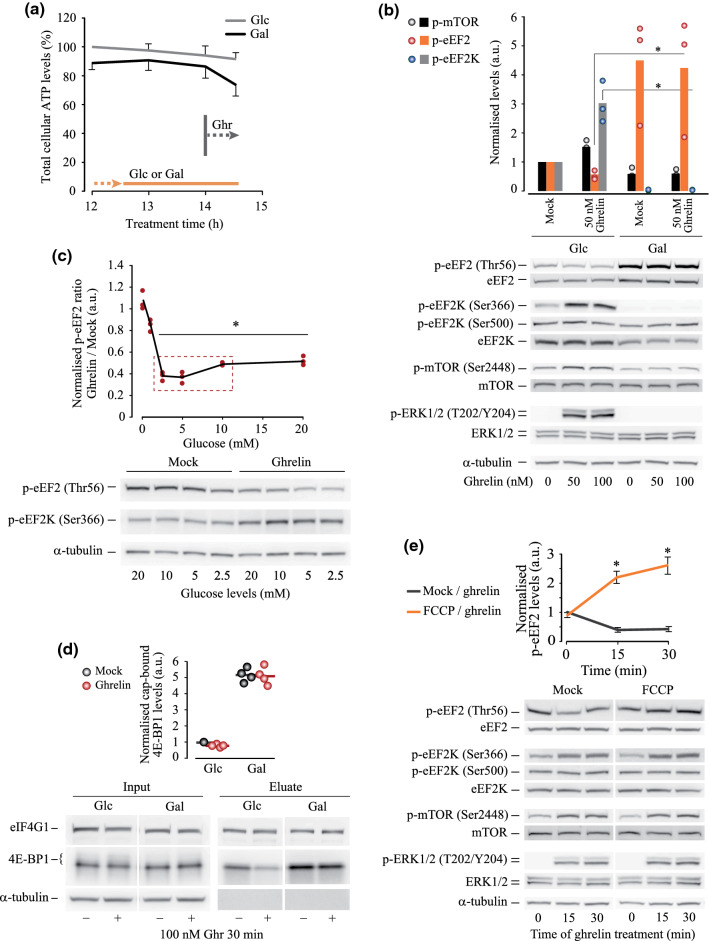
Susceptibility of ghrelin-induced de-suppression of eEF2 to metabolic stress. **a** The schematic of experiments and total ATP analysis in HEK293^GHS-R1α-EGFP+^ cells supplied with or deprived of glucose for 15 h. **b** Western blotting analysis of changes in eEF2, eEF2K, mTOR and ERK phosphorylation induced by ghrelin treatment (50 nM and 100 nM, 30 min). **c** Glucose concentration-dependent changes of p-eEF2 and p-eEF2K levels in response to ghrelin (100 nM, 30 min), Western blotting analysis. The quantification curve shows dynamics of p-eEF2 levels, and include values corresponding to 1 mM glucose concentration (see Fig. S5a, c); maximal decrease in eEF2 phosphorylation corresponding to the ‘physiological’ range of glucose concentrations (2–10 mM) is highlighted by a rectangle. **d** Levels of eIF4G1 and 4E-BP1 proteins associated with the cap binding complex in resting and metabolically stressed cells stimulated with mock (DMEM) and 100 nM ghrelin for 30 min; in glucose-deprived cells the levels of the cap-bound initiation inhibitor 4E-BP1 strongly increase. **e** Effect of ghrelin (50 nM, 15 and 30 min) on protein phosphorylation in cells pre-treated for 2 h with FCCP (mitochondrial uncoupler, 1 µM) and quantitative analysis of p-eEF2 levels. Data are presented as mean ± SD. Gradient circles demonstrate individual data points. Significant difference from mock (**c**, **e**) and between glucose (+) and galactose (−) samples (**b**) is shown by asterisks. Statistical details: **b** effect of glucose deprivation on the ghrelin-induced changes in protein phosphorylation (50 nM ghrelin), *N* = 3, independent samples *T* test: p-mTOR *t*(4) = 2.109, *p* = 0.103; p-eEF2 *t*(4) = − 3.939, *p* = 0.017; p-eEF2K *t*(4) = 5.001, *p* = 0.007. **c** Effect of glucose concentration on the response to ghrelin: (a) p-eEF2 (4,8) = 28.243, *p* < 0.001, one-way ANOVA, *p* = 0.711 for 10 mM glucose, *p* = 0.003 for 5 mM glucose,, *p* = 0.004 for 2.5 mM glucose, and *p* = 0.004 for 1 mM glucose vs 20 mM glucose, Dunnett post hoc test vs mock treatment (*N* = 3); (b) p-eEF2K (4,8) = 1.698, *p* = 0.243, one-way ANOVA, *p* = 0.90733 for 10 mM glucose, *p* = 0.76833 for 5 mM glucose, *p* = 0.57233 for 2.5 mM glucose, and *p* = 0.36799 for 1 mM glucose vs 20 mM glucose, Dunnett post hoc test vs mock treatment (*N* = 3). **d** Effect of glucose deprivation on the ghrelin-induced changes in cap-binding capacity of eIF4G1 and 4E-BP1, *N* = 4, independent samples *T* test: cap-bound eIF4G1 *t*(6) = – 1.906, *p* = 0.105; cap-bound 4E-BP1 *t*(6) = 1.132, *p* = 0.265. **e** Effect of FCCP on the response of eEF2 to ghrelin treatment, independent samples *T* test (*N* = 3): t(4) = – 15.917, *p* < 0.001 for 15 min of ghrelin treatment; *t*(4) = – 19.595, *p* < 0.001 for 30 min on ghrelin treatment

First, we examined a panel of six available antibodies expected to recognise eEF2K and different phosphorylation sites of this kinase. After the initial experiments, this panel has been shortened to three antibodies (against total eEF2K, p-eEF2K (Ser366) and p-eEF2K (Ser500)), detecting only the eEF2K-specific ~ 105 kDa bands. Using these antibodies, we found that ghrelin causes a marked increase in p-eEF2K (Ser366) levels, suggesting an involvement of Ser366 phosphorylation in the activation of eEF2 (Figs. 4b, S4). The levels of p-eEF2K (Ser366) responded to ghrelin in a time- and concentration-dependent manner. No effects of ghrelin were observed towards phosphorylation of eEF2K at Ser500 (exemplified in Figs. 5b and 6b, e). When analysing phosphorylation events downstream mTOR and ERK1/2, we found that p70 S6K and p90 RSK, two kinases known to phosphorylate eEF2K at Ser366, were activated, as evidenced by increased phosphorylation of p70 S6K (Thr389), p90 RSK (Thr573) and their target rpS6 (Ser240/244) and rpS6 (Ser235/236) [49, 50] (Figs. 4c–e, S4).

Therefore, next, we examined whether specific inhibition of rpS6 kinases could abolish the effect of ghrelin on eEF2 phosphorylation. Indeed, pre-treatment of cells with BRD7389 (an inhibitor of p90 RSK, 10 µM), PF4708671 (an inhibitor of p70 S6K, 1 µM) or a mixture of these compounds strongly reduced ghrelin-dependent changes in p-eEF2K (Ser366) and p-eEF2 (Thr56) levels (Fig. 5a–c). However, BRD7389 also decreased the total amounts of mTOR, eEF2K and eEF2 (Fig. 5b, c). With such a strong side effect of BRD7389, the involvement of p90 kinase in the ghrelin-induced Ser366 phosphorylation requires further analysis. In contrast, p70 kinase clearly modulated the effect of ghrelin on eEF2 phosphorylation. In agreement with phosphorylation data, pre-treatment of cells with PF4708671 decreased the 30-min spike in *G*Luc and SNAP protein production (Fig. 5d).

As mentioned earlier, changes in protein phosphatase PP2A activity could also have contributed to eEF2 de-phosphorylation in response to ghrelin [51]. To test this, we analysed p-eEF2 (Thr56) levels in cells pre-treated with OKA (an inhibitor of PP2A, 50 nM). To proof the efficacy of OKA, we looked at phosphorylation levels of ERK, a common target of PP2A. As expected, in the presence of OKA p-ERK1/2 levels were significantly elevated [52] (Fig. 5c). The inhibition of PP2A had no negative impact on the ghrelin-induced de-suppression of eEF2, indicating that decreased phosphorylation rather than increased de-phosphorylation drives the effect of ghrelin (Fig. 5b, c). The inhibition of PP2A caused an increase in p-eEF2K (Ser366) levels in mock-treated cells (i.e. before ghrelin addition), and an elevation of p-eEF2K (Ser366), normally induced by ghrelin, was abolished. This suggested that in the presence of OKA the response of eEF2 to ghrelin is driven by alternative mechanisms.

### Metabolic vulnerability of the effect of ghrelin on eEF2 phosphorylation

When glycolytic ATP flux is limited, cells are known to curtail energy-demanding translation. Hence, the pathways regulating eEF2K activity, including those upstream eEF2K Ser366 phosphorylation, are very sensitive to metabolic stress [38]. Therefore, we examined responses of the eEF2K/eEF2 axis to ghrelin in HEK293^GHS-R1α-EGFP+^ cells when ATP production via either glycolysis or oxidative phosphorylation (OxPhos) was compromised.

Figure 6a shows that cells deprived of glucose (supplied with l-glutamine, pyruvate and NEAA) maintained steady ATP levels through OxPhos flux. Nevertheless, the signature of ghrelin-sensitive protein phosphorylation showed high sensitivity to the inhibition of glycolysis. Thus, before addition of ghrelin, p-mTOR levels decreased, while phosphorylation of ERK, CREB and eEF2K (Ser366) was practically abolished (Figs. 6b, S5a). Glucose deprivation strongly reduced total eEF2K levels; however, the levels of p-eEF2K (Ser500) remained stable in these conditions, suggesting that relative levels of p-Ser500 and the activity of the remaining kinase molecules increased (Fig. 6b). The levels of total and phosphorylated eEF2 were strongly increased regardless of ghrelin treatment in glucose-deprived cells.

Without glucose and at low glucose levels (1 mM), treatment with ghrelin did not affect phosphorylation of eEF2 or other tested proteins with visible bands (Figs. 6b, S5a, c). Levels of p-eEF2 and p-eEF2K (Ser366) responded to ghrelin treatment when glucose concentrations ranged 2.5–20 mM, with maximal effects of ghrelin on p-eEF2 in the presence of 2.5–10 mM glucose (Figs. 6c, S5).

Levels of the cap-bound 4E-BP1 in glucose-deprived cells were strongly elevated compared to glucose ( +) control. In glucose-deprived cells, these levels were not affected by ghrelin treatment, while in cells with unlimited glycolytic flux we confirmed a negative trend in the cap binding capacity of 4E-BP1 in response to ghrelin (Fig. 6d, see also Fig. 2f). It is worth noting that although elevated m^7^GTP-bound 4E-BP1 levels in glucose-deprived cells suggested that translation initiation was inhibited, the level of m^7^GTP-bound eIF4G1 did not differ from that in cells supplied with glucose (Fig. 6d).

The observed dependence of eEF2 phosphorylation on glucose availability suggested that the response of eEF2 to ghrelin is metabolically regulated and stress susceptible. We further explored this using HEK293^GHS-R1α-EGFP+^ cells treated with a mitochondrial uncoupler FCCP as a model of mitochondrial dysfunction, which re-shapes cell bioenergetics, signalling and biosynthesis.

The uncoupling depolarises mitochondria, abolishes OxPhos, reverses F1Fo ATP synthase and bursts glycolytic flux. In our experiment, the uncoupled cells sustained ATP levels (not shown) and phosphorylation of mTOR, ERK, eEF2K and eEF2 (Fig. 6e). The effects of ghrelin on p-mTOR, p-ERK and p-eEF2K (Ser366) levels in FCCP-treated cells did not differ from that in control, while eEF2 phosphorylation in response to ghrelin increased (Fig. 6e). This suggests that the inhibitory effect of Ser366 phosphorylation on eEF2K activity was overpowered, and translation elongation was inhibited as a cell safety measure when mitochondrial function was compromised. Since Ser500 phosphorylation did not change, we hypothesised that Ca^2+^ overload driven by uncoupling was responsible for eEF2K activation via phosphorylation of residues alternative to Ser500 (Fig. 4a). Depolarised mitochondria are known to release mitochondrial Ca^2+^ and loose the capacity to shape cytosolic Ca^2+^ fluctuations [53] such as ghrelin-induced Ca^2+^ flux. Indeed, FCCP potentiated the elevation of cytosolic Ca^2+^ induced by ghrelin in HEK293^GHS-R1α-EGFP+^ cells (Fig. S3g). This suggests that when mitochondrial function is impaired, disbalanced Ca^2+^-dependent processes triggered by ghrelin could negatively regulate translation by decreasing elongation rate.

## Discussion

Protein synthesis in the cell is strictly controlled through various signalling pathways, which mediate translation initiation, elongation, termination and ribosome recycling. In this study, using HEK293, a neural cell lineage [54] overexpressing GHS-R1α, we show for the first time that GHS-R1α activation by ghrelin can rapidly accelerate protein synthesis de novo (Fig. 1a–h)*.* The concomitant decrease in Thr56 phosphorylation of eEF2, the key regulator of translation elongation rate, suggests that activated elongation facilitates/contributes to this effect (Fig. 3). We further show that a decrease in eEF2 phosphorylation is mediated through repression of eEF2 kinase activity, as a result of eEF2K (Ser366) phosphorylation by rpS6 kinases (Figs. 4, 5 and 6).

As ghrelin-mediated signalling is a complex conundrum, it is unlikely that de-suppression of eEF2 is the only event that can affect translation machinery. However, despite the activation of the ERK and PI3K/AKT/mTOR axes and downstream S6 kinases, we did not observe changes in eIF2α phosphorylation levels or eIF4F composition (Fig. 2). Levels of programmed cell death 4 (PDCD4) protein, known to inhibin cap-dependent translation, also remained unaffected by ghrelin treatment. This speaks against the contribution of mTOR/4EBP axis, a well-studied pathway regulating translation initiation, to the rapid increase in translation upon activation of GHS-R1α. While it is not clear why mTOR activation does not result in increased eIF4F availability upon ghrelin treatment, we speculate that the effect of mTOR may be counterbalanced by elevated cytosolic Ca^2+^ and AMPK signalling (Fig. 4). Being focused on eEF2 and having also examined changes in the state of eIF4F complex and eIF2α phosphorylation upon ghrelin treatment, we left eIF5A (which is considered now also a regulator of elongation and termination), eIF1 and many other initiation-related factors [55] outside the scope of this study. Based on the results of polysome profile analysis, one could expect initiation rate to increase and match the elevated elongation rate (e.g. via increased ribosome availability), thus compensating for possible changes in ribosome density (Fig. 1i, j). On the other hand, the occupancy of mRNA may not reflect changes in eEF2K/eEF2 activity, as it has been recently shown using cells with inhibited eEF2K [56]. More detailed analysis of the interplay between translation initiation and elongation is required to better understand mechanisms driving elevated protein production in response to ghrelin.

What is a putative physiological role of ghrelin-mediated effect on translation? A short-term burst in protein production might be highly relevant for specialised neuronal cells expressing ghrelin receptor. With no ATP spent producing new mRNA molecules, neurons can quickly replenish their NPY, AgRP and GABA stocks and restore synapse functionality using already existing local mRNA pool (local axonal translation is reviewed in [57]), and get ready for the next wave of ghrelin stimulation [58]. The arcuate nucleus of the hypothalamus, in which NPY mRNA levels are particularly high, is a promising model for studying ghrelin-induced changes in translation elongation and protein production in neuronal cells [59]. In addition, ghrelin ‘waves’ in the blood [2] could rhythmically increase elongation rate and favours proliferation of GHS-R1α-positive/ghrelin-negative cancer cells [31]. It is worth noting that the observed inhibitory Ser366 phosphorylation of eEF2K may limit cancer cell migration and metastasis [56], although an increased metastatic growth has been reported for a number of cancer types with the active ghrelin-GHSR axis [31]. Because of the growing evidence of the effects of ghrelin in human health and disease far beyond food intake, body weight control, energy balance and metabolism [6, 60], changes in translation are most likely implicated in these effects.

It is important to note that we conducted our study using only the n-octanoylated (C8:0 at the serin 3) form of human ghrelin, or simply ‘ghrelin’. However, other peptides of ghrelin family (e.g. d*es*-acyl ghrelin, C-ghrelin and obestatin) produced via alternative mRNA splicing and posttranslational modifications can exert specific effects or modulate effects of ghrelin on cell signalling and protein synthesis [61]. Considering that the assortment of ghrelin-related bioactive molecules is tissue specific, one could expect a broad variety of the local cell responses, including those on the level of gene expression, metabolism and cell survival [62].

There is another potential effect of eEF2 de-suppression, which may have a broad (patho)physiological consequence. Transient changes in translation elongation rates can alter translation of selected mRNAs. Over a half of human mRNAs possess upstream open reading frames (uORFs) in their 5' leader [63]. The translation efficiency of such mRNAs strongly relies on the ability of scanning ribosomes to bypass uORFs and reach main start codon, either by leaky scanning though the upstream AUGs or reinitiation (when the uORF translation is complete). Phosphorylation of eEF2 results in slowing elongation while has no known direct effect on scanning ribosomes. Therefore, it will likely affect the progression of leaky scanning ribosomes through the translated uORFs [64, 65]; this would have different impact on initiation at protein coding regions depending on the properties of uORFs in their 5' leaders. In addition, slowed elongation can promote initiation at suboptimal start codons, especially at non-AUG initiation codons such as CUG and GUG [63, 66]. Therefore, by altering elongation rates, ghrelin can change the balance between translation of canonical and alternative proteoforms in tissue-specific and context-oriented manner.

The effect of ghrelin on mRNA translation reported here does not stand alone. Other (neuro)mediators have been shown to employ eEF2 as a modulator of protein production rate. Thus, oxytocin has been shown to activate protein synthesis in eEF2-dependent manner in myometrial cells [67, 68] and hippocampal neurons [69]. Similarly, dopamine, acting through dopamine receptor D1, has been reported to elevate protein production in primary neuronal culture through the activatory de-phosphorylation of eEF2 [70]. Likewise, bidirectional changes in eEF2 phosphorylation have been observed in *Aplysia californica* (a marine mollusc) neurons in response to serotonin treatment [71], followed by alterations in the rate of protein synthesis. In concordance with the sensitivity of ghrelin effects to the ATP fluxes reported here, activation of group I metabotropic glutamate receptors (mGluRs and NMDAR) have been reported to modulate the rate of protein production in primary cortical neuronal culture in the ATP-sensitive eEF2/eEF2K-dependent manner [72].

eEF2K is considered to be a master regulator of translation, which secures metabolic adaptation of cells to nutrient deprivation [73]. In line with this, our results point to a possible role of eEF2K in nutrient and, more general, in metabolic sensing through the ghrelin signalling system. Ghrelin activates eEF2 and increases protein production rate in resting cells supplied with glucose and glutamine, both required for ATP and metabolite production. However, in our model, metabolic stress not only abolishes the role of eEF2K (Ser366) in eEF2 phosphorylation, but also prevents actual eEF2 activation by ghrelin. Mimicking common metabolic disease conditions, glucose deprivation and mitochondrial uncoupling upsurge OxPhos and glycolysis (Fig. 6). Although the outcome of the two stress conditions for eEF2 was similar, the mechanisms of their action were quite different. Glucose deficiency inhibits the activity of all tested pro-synthetic pathways, reduces total amounts and increases activity of eEF2K, abolishes Ser366 phosphorylation and strongly elevates p-eEF2 levels. With such a dominant impact of glucose deprivation in itself, ghrelin does not exert its effect and eEF2 remains hyper-phosphorylated. As a result, elongation is most likely inhibited, similar to initiation. In turn, a short-term mitochondrial uncoupling does not affect the basal levels of, as well as ghrelin-induced changes in mTOR, ERK1/2 and eEF2K (Ser366) phosphorylation (Fig. 6e). Yet, eEF2K is activated by other powerful factors evoked by ghrelin, such as overwhelmingly elevated cytosolic Ca^2+^ (see the scheme in Fig. 4a). To summarise, the effects of ghrelin on eEF2 phosphorylation and on translation elongation depend on nutrient availability and energy status of GHS-R1α-positive cells; therefore, under metabolic disease or stress conditions, ghrelin-mediated processes regulating translation elongation can be impaired or strongly affected [74].

Collectively, using an in vitro model, we demonstrate that ghrelin-induced pro-synthetic signalling cascades overpower the concomitant Ca^2+^/calmodulin-dependent inhibition of the eEF2, decrease eEF2K activity, de-suppress eEF2 and transiently activate protein production.

## Supplementary Information

Below is the link to the electronic supplementary material.

Supplementary file1 (DOCX 25321 KB)

## Data Availability

All datasets generated during this study are available by request.

## Abbreviations

AgRP: Agouti-related protein
AKT: Serine/threonine protein kinase, aka protein kinase B
AMPK: AMP-activated protein kinase
CaM: Calmodulin
cAMP: Cyclic AMP
CREB: CAMP response element-binding protein
eEF2K: Eukaryotic elongation factor 2 kinase
eIF2α, eIF4E, eIF4F, eIF4G1, IF4G2: Eukaryotic translation initiation factors 2a, 4E, 4F, 4G1, 4G2
4E-BP1: (eIF4E)-binding protein 1
ERK1/2: Extracellular signal-regulated kinases 1/2, a.k.a. mitogen-activated protein kinases (MAPK)
GABA: Gamma-aminobutyric acid
GHS-R1α: Growth hormone secretagogue receptor
GSK-3β: Glycogen synthase kinase-3β
MAPK p38δ: P38δ mitogen-activated protein kinase
MEK: Mitogen-activated protein kinase kinase
mTOR: Mammalian target of rapamycin
NPY: Neuropeptide Y
p70 S6K: P70 ribosomal S6 kinase
p90 RSK: P90 ribosomal S6 kinase
PDCD4: Programmed cell death 4
PI3K: Phosphoinositide 3-kinases
PP2A: Protein phosphatase 2A
RAF-1: Raf-1 proto-oncogene, serine/threonine kinase
rp-S6: Ribosomal protein S6
TSC1/2: Tuberous sclerosis complex proteins 1/2
