# Supplementary material for: Ghrelin rapidly elevates protein synthesis in vitro by employing the rpS6K-eEF2K-eEF2 signalling axis
Source: Cell Mol Life Sci. 2022 Jul 16;79(8):426. doi: 10.1007/s00018-022-04446-4 (PMC9288388; doi:10.1007/s00018-022-04446-4)
Supplement: Supplementary file 1 — Supplementary file1 (DOCX 25321 KB) [file 18_2022_4446_MOESM1_ESM.docx]

**Supplementary Figures**

Zhdanov A.V. et al.

Ghrelin rapidly elevates protein synthesis *in vitro* by employing the rpS6K-eEF2K-eEF2 signalling axis

**Figure S1**. **Effect of ghrelin on *de novo* production of SNAP, Gaussia luciferase (*G*Luc) and total proteins in HEK293^GHS-R1α-EGFP+^ cells**. **a** Scheme of the SNAP experiment. Cells were transfected with pcDNA3.4-SNAP plasmid and then seeded and grown on 6-well plates for 38 h, including a 14 h incubation in DMEM supplemented with 1% FBS (pre-incubations and ghrelin treatment). Cells were incubated with SNAP-Cell® Block (10 μM, 15 min), quickly washed (time ‘zero’), treated with or without ghrelin (100 nM) for indicated time and lysed with PLB (1.5 mM Mg^2+^). In the ‘Block only’ control samples, cells were lysed immediately after treatment with SNAP-Cell® Block. After lysate clarification, SNAP was stained with SNAP-Cell® 647-SiR dye (2 μM, 30-60 min at 37°C). Proteins were separated on 4-10% gel and analysed on a Typhoon Trio Imager using Cy5 settings. **b** Levels of SNAP in ghrelin (+) and ghrelin (-) cells following 15 and 30 min of ghrelin treatment; α-tubulin staining was used for loading control; N=3. **c** Effects of ghrelin on *de novo* production of *Gaussia* luciferase (*G*Luc, N=5) and SNAP (stained with SNAP-Cell® 647-SiR dye, N=4). **d** Metabolic labelling of newly produced proteins using Click-iT® HPG Alexa Fluor 594 kit: confocal analysis of Alexa Fluor 594, EGFP and NuclearMask™ Blue Stain signals in mock and ghrelin treated cells (100 nM, 30 min). Images show single planes (DIC) or stacks of seven focal planes taken with a 0.5 μm step (fluorescent dyes). Relevant quantitative analysis is shown in Fig. 1.

**Figure S2**. **Ghrelin-induced changes in protein phosphorylation in HEK293^GHS-R1α-EGFP+^ cells**: effect of treatment time (**a**) and ghrelin concentration (**b**); representative images of independent experiments. **c** Time-dependent response of CREB (Ser133 phosphorylation) to 100 nM ghrelin; (N = 1). Cells were pre-incubated for 12-16 h in serum-free DMEM supplemented with NEAA and then treated with ghrelin as indicated. Data were normalised to the corresponding total protein levels.

**Figure S3**. **Effect of ghrelin on GHS-R1α internalisation and cytosolic Ca^2+^ levels in HEK293^GHS-R1α-EGFP+^ cells**. Live cell confocal imaging analysis. **a-c** Ghrelin-induced changes in GFP intensity across representative cells, a line profile analysis (arrows show cell borders) and ratios between cytosolic- and plasma membrane- (PM) associated GFP signals before and 30 min after addition of ghrelin. **d** Changes in OGB-1 (Ca^2+^ indicator) lifetime (LT) upon ghrelin treatment, confocal FLIM analysis. Images produced by SPCImage software show LT of the dye as rainbow false colours. OGB-1 LT increase and colours change from ‘cold’ to ‘warm’ upon binding of OGB-1 with Ca^2+^. **e** LT images of GFP alone (no OGB-1 staining), shown with the same colour settings as in (**d**). **f** Histograms of GFP and OGB-1 LT distribution in fields of view during ghrelin treatment; right panel shows cumulative LT histogram, from which the 25^th^, 50^th^ and 75^th^ percentile values can be calculated (dotted lines). An increase in these values report on the elevation of Ca^2+^ levels. **g** Ca^2+^ levels in cells treated by FCCP (1 μM) and ghrelin (30 min, 50 nM, see Fig. 6e). Confocal FLIM analysis of OGB-1 probe is shown as cumulative LT distribution histogram.

In all experiments (N=3), cells were pre-incubated in DMEM supplemented with 1% FBS for 12-14 h and then treated with 100 nM ghrelin. Fluorescence images are stacks of three focal planes taken with 0.5 μm steps (**a**) or three focal planes taken with 1 μm steps (**d**).

*Statistical details:*

(d) Receptor internalisation, independent samples T test, N=3. T(24)=-17.164, p<0.001.

**Figure S4**. Time- and concentration-dependent effects of ghrelin on protein phosphorylation in HEK293^GHS-R1α-EGFP+^ cells (representative independent experiments, Western blotting analysis). **a** Effect of treatment time. **b** Effect of ghrelin concentration. **c** Quantitative analysis of p-rpS6 levels; data are presented as mean±SD. Cells were pre-incubated for 15-16 h in serum-free DMEM supplemented with NEAA.

*Statistical details:*

(c*) Effect of ghrelin treatment time:* p-rpS6 (4,16) = 150.75, p<0.001, one-way ANOVA, p<0.001 for 15, 30, 45 and 60 min, Dunnett post hoc test vs mock treatment (N=5). G*hrelin concentration effect:* p-rpS6 (4,16) = 79.086, p<0.001, one-way ANOVA, p=0.002 for 15 min, p=0.01 for 30 min, p<0.001 for 45 and 60 min, Dunnett post hoc test vs mock treatment (N=5)

**Figure S5**. Effect of ghrelin on eEF2 (Thr56) and eEF2K (Ser366) phosphorylation in HEK293^GHS-R1α-EGFP+^ cells at different glucose concentrations. Cells were pre-incubated in DMEM supplemented with 1% FBS (dialysed to remove glucose) and 0-20 mM glucose for 14 h and then treated with ghrelin (100 nM, 30 min). a Glucose concentration-dependent dynamics of p-eEF2 and p-eEF2K levels in response to ghrelin. b, c Representative biological replicates of the effects of glucose concentrations on responses of eEF2 and eEF2K to ghrelin treatment. Maximal kinase activity is seen in glucose-deprived cells, in which the total eEF2K levels are strongly reduced (4-7-fold). In (a), p-ERK is used as a positive control of the response to ghrelin; α-tubulin levels serve for data normalisation.
